# PERMA+4: A Framework for Work-Related Wellbeing, Performance and Positive Organizational Psychology 2.0

**DOI:** 10.3389/fpsyg.2021.817244

**Published:** 2022-01-24

**Authors:** Stewart I. Donaldson, Llewellyn Ellardus van Zyl, Scott I. Donaldson

**Affiliations:** ^1^Division of Behavioral and Organizational Sciences, Claremont Graduate University, Claremont, CA, United States; ^2^Department of Industrial Engineering and Innovation Sciences, University of Eindhoven, Eindhoven, Netherlands; ^3^Optentia Research Unit, North-West University, Vanderbijlpark, South Africa; ^4^Department of Human Resource Management, University of Twente, Enschede, Netherlands; ^5^Department of Social Psychology, Institut für Psychologie, Goethe University, Frankfurt, Germany; ^6^Department of Population and Public Health Sciences, Keck School of Medicine, University of Southern California, Los Angeles, CA, United States

**Keywords:** PERMA+4, wellbeing, work-related wellbeing, positive organizational psychology, future perspectives, work performance

## Abstract

A growing body of empirical evidence suggests that positive emotions, engagement, relationships, meaning, and accomplishments (PERMA) may be a robust framework for the measurement, management and development of wellbeing. While the original PERMA framework made great headway in the past decade, its empirical and theoretical limitations were recently identified and critiqued. In response, Seligman clarified the value of PERMA as a framework for and not a theory of wellbeing and called for further research to expand the construct. To expand the framework into organizational contexts, recent meta-analyses and systematic literature reviews showed that physical health, mindset, physical work environments and economic security could be seen as essential contextually relevant building blocks for work-related wellbeing and are therefore prime candidates to expand the PERMA framework for use within organizational contexts. Through expanding the original PERMA framework with these four factors, a new holistic approach to work-related wellbeing and work performance was born: the PERMA+4. As such, the purpose of this brief perspective paper is to provide a conceptual overview of PERMA+4 as holistic framework for work-related wellbeing and work performance which extends beyond the predominant componential thinking of the discipline. Specifically, we aim to do so by providing: (1) a brief historical overview of the development of PERMA as a theory for wellbeing, (2) a conceptual overview of PERMA+4 as a holistic framework for work-related wellbeing and work performance, (3) empirical evidence supporting the usefulness of PERMA+4, and (4) charting a course for the second wave of positive organizational psychological research.

## Introduction

Positive psychology has emerged as one of the most rapid-growing sub-disciplines in psychology (Martín-del-Río et al., 2021). During its first decade as a stand-alone science, positive psychological research has grown to account for 4% of all research conducted and published in psychology (Rusk and Waters, 2013). In their bibliometric analysis, Rusk and Waters (2013) found that positive psychological research spanned the full range of psychological sub-disciplines ranging from sport- to clinical psychology. However, most publications (18.74%) seemed to be related to positive psychology *at work* (categorized as “management psychology” −10.88% and “business” −7.68%; Rusk and Waters, 2013). The popularity of positive psychology at work has since increased exponentially within the literature, with around 5,880 manuscripts (totaling 66,635 citations; Martín-del-Río et al., 2021). This groundswell of interest into understanding, measuring, managing and developing positive aspects of work is aptly labeled “Positive Organizational Psychology” (POP; Donaldson and Ko, 2010).

POP has been defined “as the scientific study of positive subjective experiences and traits in the workplace and positive organizations, and its application to improve the effectiveness and quality of life in organizations” (Donaldson and Ko, 2010, p. 177) and draws from the developments in positive organizational behavior (Luthans, 2002) and positive organizational scholarship (Cameron et al., 2003). POP aims to apply the scientific method to investigate the positive states, − traits and – behaviors associated with work-related wellbeing and work performance, which, in turn, spawned a myriad of new theories (e.g., Appreciative Inquiry), constructs [e.g., Psychological Capital (PsyCap)], measuring instruments (e.g., Team flow index) and approaches to organizational interventions (e.g., Positive Psychological Coaching; van Zyl et al., 2020; Richter et al., 2021). These new (positive) approaches towards work-related wellbeing, and work performance has shown to be better predictors of individual and organizational performance than the Big Five personality dimensions, cognitive abilities, emotional intelligence, the situational judgment test, interviews, and in-basket tests (c.f. Moscoso and Salgado, 2021).

Despite these advances and findings, the POP’s approach toward measuring, managing and developing work-related wellbeing and work performance has faced a significant amount of criticism (Wong and Roy, 2017; van Zyl and Rothmann, 2019; Goodman et al., 2020). First, critics argue that POP constructs suffer from the “jangle fallacy,” where old psychological constructs are merely redressed in new “jackets” to see novel/innovative but are fundamentally still the same (Brown et al., 2014; Compton and Hoffman, 2019; Yakushko, 2019). For example, Duckworth’s (2016) “Grit” is seen as indistinguishable from conscientiousness and/or mere perseverance (van der Vaart et al., 2021). Second, positive psychological assessment measures produce inconsistent factorial structures, varying levels of internal consistency, are culturally biased and produce questionable levels of predictive validity (van Zyl and Ten Klooster, 2022). For example, the Mental Health Continuum Short Form and the Grit Scale has been shown to produce no less than 10 different factorial structures, with varying levels of internal consistencies across cultures (van Zyl and Ten Klooster, 2022). Third, positive organizational interventions do not produce significant nor sustainable changes in wellbeing and where significant changes are shown, they are small or marginal at best (Wong and Roy, 2017). For example, two recent systematic literature reviews of brief positive psychological interventions, Ivandic et al. (2017) and Roll et al. (2019) found limited evidence of the effectiveness to reduce negative work-related experiences. Fourth, POP relies too heavily on “contextual factors” to argue or justify non-replicable results (Parks and Schueller, 2014; Friedman and Brown, 2018). For example, in various job crafting interventions, no positive effects on outcome factors could be found. In each study the authors argue that contextual factors (such as the implementation of a new system, organizational restructuring or the environment) played a role in explaining why the intervention was ineffective (c.f. Demerouti et al., 2019; Hulshof et al., 2020). Critics argue that this is due to poorly defined grand theories and a lack of an overarching metaparadigm/metatheory, where unexpected results (that deviate from hypotheses) are defended rather than explored and theories updated (Friedman and Brown, 2018; Hughes, 2018).

Finally, critics argue that POP lacks a unifying metatheory and a series of grand theories or frameworks that explain the development of holistic wellbeing (Wong and Roy, 2017; Friedman and Brown, 2018; Joseph, 2021). Without a unifying metatheory, positive organizational researchers will be confined to componential thinking whereby the focus is on understanding a specific state-, trait- or behavior outside of its context and in isolation of other factors. Metatheories focus on broad and paradigmatic issues related to general theory development in a new discipline (e.g., the purpose of theories and what types of theories are needed, proposing and criticizing criteria for theory development and evaluation) and are comprised of a series of increasingly restrictive grand theories, middle-range theories, and theoretical models (Wallis, 2010). In their seminal work, Seligman and Csikszentmihalyi (2000, p. 5) attempted to provide a meta-theoretical framework for positive psychology by arguing that such is “a science of positive, subjective experience, positive individual traits and positive institutions [aimed at] improving quality of life and to prevent the pathologies that arise when life is barren or meaningless.” However, their manuscript failed to outline the purpose of positive theories, which types of theories are needed, and the criteria used to evaluate “positive” theories. It also failed to provide the methods or processes required to generate knowledge. Therefore, their initial conceptualization does not meet the criteria for a metatheory or metaparadigm but could instead be seen as a Grand Theory of general psychology.

On the other hand, grand theories are highly abstract where the focus is more on the formal organization and arrangement of the concept, rather than explaining or understanding social reality (Skinner, 1990). Grand theories are too abstract to state the nature or direction of the relationships between factors in empirical terms or to specify actions or processes for practice. With the exception of Self-Determination Theory (Ryan and Deci, 2000), and the elements borrowed from Existentialism (Wong, 2012), humanistic psychology (Joseph, 2021) and others, grand theoretical approaches that provide an interpretative framework for the formal organization of a phenomenon in positive psychology is lacking. Although various approaches such as Strengths-Theory (Peterson and Seligman, 2004), the Broaden-and-Build theory on positive emotions (Fredrickson, 2001), and the PERMA model for human flourishing (Seligman, 2011) are positioned as “grand theories,” they lack the capacity to explain the organization of complex phenomena and are too narrow and specific in focus.

For example, Seligman’s (2011) PERMA approach towards wellbeing “is not a formal theory, but rather a listing of the phenomena that have been shown to [only] be related to wellbeing” (Wong and Roy, 2017, p. 142). Seligman (2011, p. 13) argued that wellbeing is a function of Positive Emotions, Engagement, Relationships, Meaning and Accomplishments and that PERMA should be considered “the gold standard for [understanding] wellbeing.” Within organizational contexts, Slavin et al. (2012) argued that the PERMA model should be seen as a functional model for facilitating institutional leadership and to create positive organizational culture. Yet no theoretical argument underpinning these factors as components, rather than mere correlates of wellbeing, was provided (van Zyl, 2013; Wong and Roy, 2017). Further, the PERMA approach negates other factors known to be essential to work-related wellbeing such as the impact of the work or physical environment (Lyubomirsky et al., 2005), positive physical health (Seeman, 1989), growth mindsets (Dweck and Yeager, 2019) and economic prosperity (Biswas-Diener and Patterson, 2011; Ng et al., 2021). Similarly, Goodman et al. (2017) found that PERMA does not attribute any unique variance in wellbeing when compared to other types of wellbeing indicators. Therefore, PERMA is too narrow in scope and does not provide a clear set of propositions about how or why these concepts relate nor does it provide theoretical justification for its position within the broader nomological network of POP (Goodman et al., 2017; Kashdan, 2017). PERMA may therefore be redundant or arbitrary as a measure of both general- (Kashdan, 2017) and work-related wellbeing (Donaldson, 2019). As such, PERMA does not meet the criteria of a grand theory, nor a midrange theory of wellbeing. But rather be seen as a base model for understanding the elements or “building blocks” leading to work-related wellbeing and work performance (Seligman, 2008).

Although it is beyond the scope of this brief paper to reflect upon each of the criticisms, we believe that the final critique is the most important and that addressing such would, by virtue, affect the other challenges. Therefore, a more holistic approach towards work-related wellbeing and work performance is needed by expanding upon the routes to or elements of the construct. Such an approach would provide the discipline with a means to develop and grow, and provide practitioners with a holistic framework on which to assess and develop wellbeing at work. As such, the purpose of this brief perspective paper is to provide a holistic theoretical framework for work-related wellbeing and work performance which extends beyond the predominant componential thinking of the discipline. We do this through providing: (1) a brief historical overview of the development of PERMA as a theory for wellbeing, (2) a conceptual overview of PERMA+4 as a holistic framework for work-related wellbeing and work performance, (3) empirical evidence supporting the usefulness of PERMA+4, and (4) charting a course for the second wave of positive organizational psychological research.

## Building Blocks of Wellbeing

Wellbeing and positive functioning are considered essential elements for developing sustainable work performance (Donaldson and Ko, 2010). Wellbeing is seen a state in which an employee “realizes his or her own abilities, can cope with the normal stresses of life, can work productively and fruitfully, and can contribute to his or her community” (World Health Organization, 2004, p. 2). Although various competing approaches to work-related wellbeing exist within the literature, all share the same fundamental principle: to help people fit in and function well at work (Rothmann, 2013). While the “fitting in” component can be controlled for during the recruitment and selection process (by ensuring a good person-job, person-team, and person-organization fit), the “functioning well” component is more important to ensure sustainable work performance (Donaldson et al., 2021; Donaldson and Donaldson, 2021a). Functioning well or “Positive functioning” at work refers to a combination of an employee’s positive emotional experiences at work (hedonic wellbeing) and the factors needed to perform optimally in one’s work role (eudemonic wellbeing; Rothmann, 2013). In other words, positive functioning occurs when individuals are able to effectively manage the daily fluctuations in positive- and negative emotions at work (i.e., affect balance) and having the opportunity to live up to their potential, having a sense of meaning/purpose at work, harboring feelings of control over one’s work-life and the execution of ones duties and being able to build and maintain positive work-related relationships (van Zyl and Rothmann, 2014). This, in turn, leads employees to perform better at work related tasks and leads to extra-role performances (e.g., organizational citizenship behaviors; Albrecht, 2012; Sulea et al., 2012; Davila and Finkelstein, 2013; Warr and Nielsen, 2018). Therefore, positive functioning is an integral part of overall work-related wellbeing and is strongly associated with work performance (Donaldson, 2019; Donaldson et al., 2019). It is therefore not surprising that many POP interventions aim to enhance employees’ work-related wellbeing as a means to increase their work performance (Roll et al., 2019; Donaldson and Chen, 2021). However, there is still no consensus on the exact elements or “building blocks” of wellbeing that should be targeted to sustainably enhance work performance (Seligman, 2008; Donaldson and Chen, 2021).

One approach that could provide a roadmap for sustainable performance through wellbeing is PERMA (Seligman, 2011). The PERMA model was positioned as an extension of Seligman’s (2002) original theory of authentic happiness. Seligman (2002) argued that happiness is the result of an integration between two philosophical conditions: hedonism (pursuing pleasure and avoiding pain) and eudaimonia (living in accordance with one’s own daimon). Drawing from these two traditions, Seligman (2002) defined happiness as a positive psychological state characterized by three building blocks: pleasure (“pursuing positive- and avoiding negative emotions”), meaning (“experiences where one is connected to something larger than the self”), and engagement (“experiences where one is absorbed or fully cognitively/physically/emotionally emerged in one’s hobbies/work/life”). In the original empirical investigation of authentic happiness theory, Peterson and Seligman (2004, p. 40) concluded that “these orientations are distinguishable, that they are not incompatible and thus able to be pursued simultaneously, and that each is associated with life satisfaction.” This implies that these three building blocks are independent (yet related) that they can be pursued independently of one another (Peterson and Seligman, 2004) and that these can actively be developed through interventions (Seligman, 2011). However, pursuing these three factors alone is not enough to ensure sustainable changes in wellbeing (Seligman, 2011). As such, Seligman (2011) argued that for authentic happiness to lead to overall wellbeing, it requires two additional components: building and maintaining positive relationships and through accomplishments. This extension of authentic happiness theory, by including positive relationships and accomplishments, led to Seligman’s (2011) new theory called “PERMA.”

So what, according to Seligman (2011), is PERMA? Seligman (2011) argued that PERMA is not as a theory *of* wellbeing but should rather be considered as framework *for* wellbeing (Seligman, 2008). In other words, PERMA does not describe what wellbeing is, but rather provides a framework for the routes or building blocks to consider when one wants to develop wellbeing. In effect, Seligman (2011) stated that wellbeing can actively be develop through pursuing five measurable elements, which he called PERMA:

1. **Positive emotions**. Experiencing happiness, joy, love, gratitude, etc. in the here and now
2. **Engagement**. Being highly absorption, emersed or experiencing flow while engaged in activities of one’s life
3. **Relationships**. Having the ability to establish and maintain positive, mutually beneficial relationships with others characterized by experiences of love and appreciation
4. **Meaning**. The experience of being connected to something larger than the self or serving a bigger purpose.
5. **Accomplishment**. Experiencing a sense of mastery over a particular domain of interest or achieving important or challenging life/work goals.

Individually, these elements were found to be highly predictive of wellbeing and within work-related contexts showed strong associations with work performance (c.f. Donaldson and Donaldson, 2021a). However, as mentioned before, the PERMA model is not without critiques, some of which have already been discussed (c.f. Donaldson et al., 2020 for a more extensive exposition on the topic). Seligman (2008) strongly disagreed with the criticisms and affirmed PERMA as a framework of elements required *for* wellbeing instead of a theory of what wellbeing is. He argued that these elements are not exhaustive but acknowledged that additional evidence-based building blocks might improve the framework. Albeit not being exhaustive, PERMA is exclusive and specific criteria should be considered when considering the expansion of the construct (Seligman, 2008). Seligman (2008) then set six specific criteria researchers should consider before introducing new components:

1. New elements should show to directly and positively relate to wellbeing,
2. Individuals should pursue each new element for its own sake, and not in service or pursuit of another,
3. PERMA should be seen as an exclusive, yet not exhaustive framework that is open and flexible for new developments,
4. New elements should lead to specific developmental interventions aimed at enhancing wellbeing,
5. The list of factors should at all times be parsimonious, and
6. Each new element should be independently defined and measured in relation to others.

Anecdotally, with these six criteria, Seligman (2008) addressed a number of the criteria underpinning the creation of robust theories: clarifying the purpose of the theory (through highlighting that it is an approach to rather than of wellbeing), highlighting what additional types of approaches/elements are needed for its expansion, setting specific criteria for theory development and evaluation and inviting further theorizing (Wallis, 2010). Thus, providing a solid basis for further theory building.

## A Holistic Approach to Wellbeing at Work: The PERMA+4 Framework

In his conclusion, Seligman (2008) encouraged the scientific community to search for additional building blocks which may enhance or strengthen the PERMA framework. With more than two decade’s worth of empirical research underpinning the relationship between the individual elements of PERMA and other forms of wellbeing, this approach could act as a foundational base from which to build a more holistic framework work-related wellbeing and sustainable work performance (Seligman, 2008 Kern et al., 2014 Kern et al., 2015a,b; Bulter and Kern, 2016). As such, based on Seligman’s (2008) fourth criteria, Donaldson (2019) and Donaldson et al. (2020) conducted an extensive systematic literature review, meta-analysis, and a range of qualitative assessments in order to determine if and how the framework could be extended into work-related contexts. Their main aim was to determine which additional elements seemed likely to contribute to work-related wellbeing and sustainable work performance over and above the original five elements (Donaldson et al., 2020). They found that four additional building blocks could explain additional variance in work-related wellbeing and work performance and could thus be considered for inclusion into the PERMA framework. Donaldson (2019), Donaldson and Donaldson (2021a,b), and Donaldson et al. (2020) found empirical evidence supporting the addition of these four elements:

1. **Physical Health**. Operationalized as a combination of high levels of biological, functional, and psychological health assets.
2. **Mindset**. Adopting a growth mindset characterized by an optimistic, future-oriented view of life, where challenges or setbacks are seen as opportunities to grow. This may also be a function of psychological capital, perseverance or grit.
3. **Work Environment**. The quality of the physical work environment (which includes spatiotemporal elements, such as access to natural light, fresh air, physical safety and a positive psychological climate) aligned to the preferences of the individual
4. **Economic Security**. Perceptions of financial security and stability required to satisfy individual needs.

### Physical Health

One of the main criticisms of work-related wellbeing interventions is that they negate the importance of physical health as part of the developmental process (Biddle et al., 2021). This is somewhat surprising because a substantial amount of literature (ranging from medical sciences to anthropology) has shown that physical health is one of the most essential components of wellbeing and mental health (Biddle et al., 2021). Seligman (2008) argued that positive physical health is an essential element that buffers against the onset of psychological disorders and is integral to psychological wellbeing. Positive physical health is conceptualized as state of optimal physiological functioning, which is more than just the absence of disease or infirmity (World Health Organization, 2004, p. 10). In essence, positive physical health aims to promote individuals’ positive health assets: (1) biological assets, (2) functional assets and (3) subjective or psychological health assets. *Biological assets* refer to the positive ends of one’s physiological or anatomical functioning such as physical fitness, health body-mass index, heart-rate variability, pulse, blood pressure (Seeman, 1989). Donaldson and Donaldson (2021a,b) also postulate that biological assets may include mindful reflection on one’s own personal health history or health habits.

In contrast, *functional assets* refer to how well individuals can function in the execution of their physical duties in life or at work (Seligman, 2008). This may include self-reported reflections on physical activity or fitness at work (Donaldson and Donaldson, 2021a,b). The final asset pertains to “subjective” or psychological health assets, which is fundamentally a function of how one feels. Here the focus is on aspects that enhance perceptions of physical health, such as a sense of dedication, vigor, absorption, or vitality when engaged in physical activity (van Berkel et al., 2013; Seligman, 2008). Similarly, it pertains to the absence of subjectively perceived health complaints (such as aches and pains), a sense of durability or confidence about one’s body, a feeling of control over health-related matters, optimism about longevity and future health, and high levels of overall life satisfaction (Jackson, 2007; Seligman, 2008; Ng et al., 2021). Physical health can also be developed at work and has been shown to effectively supplement the effects of more traditional work-related wellbeing programs (Biddle et al., 2021). The main point though is that within an individual’s range of possible physical health levels, those that learn to function at the high end of their range are more likely to feel and function well.

### Mindset

Those who hold the belief that their talents can be developed through hard work and deliberate practice (i.e., holding a growth mindset) usually report higher levels of wellbeing and performance than those who view their talents to be innate or fixed (i.e., holding a fixed mindset; Dweck and Yeager, 2019). Holding a growth mindset is characterized by the belief that one’s intellectual abilities and talents are malleable and can be developed over time (Tang et al., 2019). Individuals with a growth mindset tend to choose more challenging tasks that help stretch their current capabilities to facilitate personal growth and development (van Zyl et al., 2021). These individuals tend to see failures as opportunities to grow and are more likely to dissect mistakes in order to avoid similar situations in the future (Tang et al., 2019). In contrast, those with a fixed mindset attribute failures and successes to external factors and are more likely to shy away from challenges or fail to live up to their potential (Dweck, 2008). At work, those with a growth mindset tend to invest in their personal development (Caniëls et al., 2018), actively seek feedback on their performance to improve and show a mastery orientation to goal attainment (van Zyl et al., 2021). Further, those who hold a growth mindset at work should also show positive beliefs that their work will provide them with opportunities to grow, that they can meaningfully contribute to the goals of the organization and that work will provide meaningful challenges to test and stretch their capabilities (Donaldson et al., 2020; Donaldson and Donaldson, 2021a,b; van Zyl et al., 2021). It is, therefore not surprising that growth mindset interventions at work have shown to have a significant effect on positive individual (e.g., mental health; wellbeing; and engagement) and organizational outcomes (e.g., increased performance; Han and Stieha, 2020).

In more context-specific terms, PsyCap could be seen as another indicator or element of building a positive mindset at work (Luthans and Youssef-Morgan, 2017; Luthans and Broad, 2019; Donaldson et al., 2021; Youssef-Morgan et al., 2021). Psychological capital refers to the development-orientated mindset individuals adopt that is characterized by “(1) having confidence to take on and put in the necessary effort to succeed at challenging tasks (self-efficacy), (2) making a positive attribution about succeeding now and in the future (optimism), (3) persevering toward goals and when necessary, redirecting paths to goals in order to succeed (hope), and (4) when beset by problems and adversity, sustaining and bouncing back and even beyond to attain success (resilience)” (Luthans et al., 2015, p. 2). More recently, Youssef-Morgan et al. (2021) argued that work-related gratitude should be seen as an integral (additional) component of PsyCap. They argued that work-related gratitude is an “the intentional choice to engage in positive appraisals and feelings of thankfulness and appreciation toward the characteristics, situations, and people currently present in one’s work context. Specifically, this definition synthesizes the conative (intentional choice), cognitive (positive appraisals), affective (feelings), and social (people) aspects of gratitude. Further, it takes into consideration that gratitude is a situational and context-specific state, rather than just a general disposition” which complements and supports PsyCap theory (Youssef-Morgan et al., 2021, p. 3). These factors are considered personal or psychological resources that synchronously interact to produce a development-based mindset overtime through intentionality, goal pursuit and self-discipline (Luthans and Youssef-Morgan, 2017). Hope, self-efficacy, work gratitude, and optimism are proactive in nature, and resilience re-active (Luthans et al., 2015). This implies that PsyCap not only buffers against negative experiences associated with goal pursuits (i.e., resilience), but also facilitates goal attainment through framing failures/opportunities as positive stepping stones or growth opportunities (Donaldson et al., 2021).

PsyCap has shown to be an integral component for facilitating individual and organizational performance and to enhance wellbeing (Donaldson et al., 2020). Donaldson et al. (2020) also argued that PsyCap is not a static trait, but also a state which could actively be developed through human resource development practices and interventions. Salanova and Ortega-Maldonado (2019) demonstrated that interventions aimed at creating a positive mindset through PsyCap are effective, sustainable, durable, cross-culturally impactful and integral for enhancing work-related wellbeing. Given that PsyCap is state-like and malleable, as well as future-focused and associated with wellbeing and work performance, it seems to be an important factor to consider in the expansion of PERMA.

### Work Environment

The physical work environment of employees can significantly impact both their physical health and wellbeing (Boegheim et al., 2021; Bergefurt et al., 2022). Given that individuals spend more than a third of their lives at work or engaged in work-related activities, Sander et al. (2019) argued that the physical working environment may be one of the biggest contributors to wellbeing and performance at work. The physical work environment consists of all objects, stimuli and subjective evaluations of organizational climate/culture that employees encounter through the execution of their work roles at work (Bergefurt et al., 2022). The work environment is therefore seen as a complex psychophysical system which is a function of bot the objective physical stimuli at work (e.g., building design, air quality, and natural lighting) but also elements subjectively experienced by employees (e.g., perceptions of physical safety or connectedness to others; Sander et al., 2019).

Sander et al. (2019) argued that wellbeing and performance at work are influenced by their cognitive, affective, and relational responses to the whole office environment. Cognitive reactions refer to the extent towards which the physical work environment affords individuals the opportunity to concentrate on their relevant tasks (i.e., Focus; Sander et al., 2019). Focus is considered the most fundamental element of performance and can directly be influenced by the physical environment. When there is considerable effort required to focus due to environmental distractions (such as noise, heating or poor ventilation) cognitive resources are depleted thus increasing stress and strain (Veitch, 2018). Affective reactions incorporate mood and emotions and pertain to non-cognitive responses to the physical design of the work environment (i.e., Sense of Beauty; Sander et al., 2019). This, in turn, may have a restorative function on employees’ energies (Nasar, 1997). When individuals perceive a sense of beauty at work (whether it be due to the design of the office or access to nature), they are more likely to experience positive affect. White (1996) argued that perceptions of beauty at work are essential to foster positive at work. Further, from the psychological strengths perspective, “appreciation of beauty” has also been shown to increase wellbeing and esthetically pleasing organizations fosters a sense of trust in the company (Peterson and Seligman, 2004; Proyer et al., 2016). Finally, relational reactions refer to the effect of the physical environment on creating or fostering a connection between people (Sander et al., 2019). For example, if individuals are located in different buildings (or floors) in the same organization, yet working in the same team, they are less likely to engage with each other physically (Sander et al., 2019; Bergefurt et al., 2022). In essence, the physical work environment directly affects with whom and how often people connect or interact at work, and it may influence the relationships element of PERMA+4 as well. Therefore, relational reactions are a function of the connectedness the work environment fosters (Boegheim et al., 2021; Bergefurt et al., 2022). These three factors have shown to directly and significantly impact overall experiences of wellbeing (both positively and negatively; Boegheim et al., 2021; Bergefurt et al., 2022). Workplace design interventions can therefore play a significant role in not only enhancing productivity but also facilitate wellbeing (Sander et al., 2019; Boegheim et al., 2021; Bergefurt et al., 2022).

### Economic Security

Recent research using advanced machine learning approaches, which maximize prediction by thoroughly exploring nonlinear effects and higher-order interactions, has found that one’s control over financial matters is one of the strongest predictors of wellbeing (Margolis et al., 2021). The ninth building block in the PERMA+4 framework is economic or financial security (also referred to as financial wellbeing in alternative literature). Economic security refers to the impact one’s level of income, savings, and spending has on wellbeing (Zemtsov and Osipova, 2016; Donaldson and Donaldson, 2021a,b). Salignac et al. (2020) argued that making sound financial decisions and exerting control over financial matters are pertinent to overall wellbeing. If one is not able to meet basic physiological needs (such as purchasing food for dinner) or unable to attend to financial obligations (e.g., paying debts, school fees, or medical bills), it may lead to increases in stress, depression and anxiety (Salignac et al., 2020). Those with extreme debt who cannot manage these obligations are more likely to report suicide attempts than those without debt (Naranjo et al., 2021; Rojas, 2021). In contrast, if there is relative certainty about one’s financial future, individuals are able to more effectively plan and make bigger life decisions (such as having children or purchasing a house; Rojas, 2021). This, in turn, also creates surety and stability (Rojas, 2021). Although economic security cannot actively be developed, planning, managing, and controlling spending behavior can. Studies have shown that interventions aimed at training basic financial literacy and financial planning directly impact happiness, health and wellbeing (Lowe et al., 2018).

**Figure 1:**
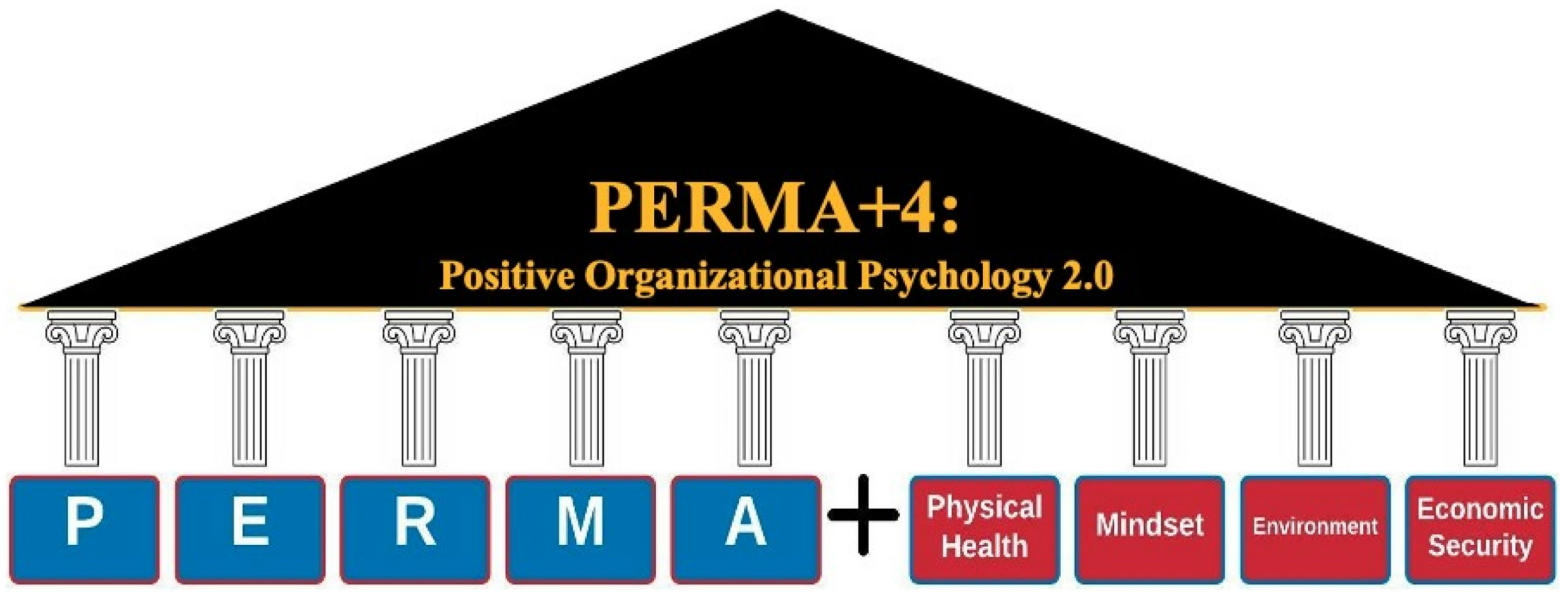
The PERMA+4 framework.

Despite these factors’ relative importance to work-related wellbeing and work performance, these four factors should be tested against Seligman’s (2008) criteria before they can be considered for inclusion. Through this brief conceptual overview of the additional four components, we highlighted that each component is positively and directly associated with wellbeing, that each element is pursued for the sake of itself and not a function of another, that interventions are already available targeting each element, that the addition of these elements do not distract from the parsimonious nature of PERMA and that each element is independently measured and defined (c.f. Table 1). As such, these four elements can confidently be incorporated into the PERMA framework as a means to expand such into organizational contexts. Given that all Seligman’s (2008) criteria are met, these four factors can be included into the expansion of PERMA: thus giving birth to the PERMA+4 (c.f. Figure 1).

**Table 1:** New building blocks and Seligman’s criteria.

	Seligman’s Criteria	Physical Health	Mindset	Work Environment	Economic Security
1	Positively and directly related to wellbeing	Yes	Yes	Yes	Yes
2	Pursing elements for its own sake	Yes	Yes	Yes	Yes
3	Interventions available aimed at new element’s development	Yes	Yes	Yes	Indirectly
4	Adds to Parsimony	Yes	Yes	Yes	Yes
5	Element is independently measured and defined	Yes	Yes	Yes	Yes

## Empirical Findings Supporting PERMA+4

The PERMA+4 framework has also been subjected to some empirical investigation. First, Donaldson (2019) and Donaldson and Donaldson (2021b) developed and evaluated the Positive Functioning at Work (PFW) Scale, which aimed to measure the nine building blocks of the PERMA+4 model. The PFW is a 29 item self-report measure that aims to measure the nine building blocks of wellbeing (c.f. Table 2). The results showed that both a nine first-order factorial model, as well as a hierarchical second-order model (comprised of nine first-order factors), fitted the data well and exhibited convergent, discriminant, criterion, predictive, and incremental forms of validity with other forms of wellbeing (Satisfaction with Life: Diener et al., 1985; PsyCap: Luthans et al., 2007) and performance measures (Positive Work Role Performance: Griffin et al., 2007), as well as measurement invariance across job function (Donaldson and Donaldson, 2021a,b).

**Table 2:** Measuring PERMA+4: the positive functioning at work scale.

Dimension	Sub-Dimension	Items	Label
**Positive Emotions**	Future-Oriented and Affective	1. I feel joy in a typical workday	P1
		2. Overall, I feel enthusiastic about my work	P2
		3. I love my job	P3
**Engagement**	Absorption	4. I typically become absorbed while I am working on something that challenges my abilities	E1
		5. I lose track of time while doing something I enjoy at work	E2
		6. When I am working on something I enjoy, I forget everything else around me	E3
**Relationships**	Giving	7. I can receive support from coworkers if I need it	R1
	Perceived	8. I feel appreciated by my coworkers	R2
	Shared Compassion	9. I trust my colleagues	R3
	Psychosocial	10. My colleagues bring out my best self	R4
**Meaning**	Transcendent	11. My work is meaningful	M1
	Meaning	12. I understand what makes my job meaningful	M2
	Greater Good Motivations	13. The work I do serves a greater purpose	M3
**Accomplishment**	Goals	14. I set goals that help me achieve my career aspirations	A1
		15. I typically accomplish what I set out to do in my job	A2
	Prove (Performance Goal) Orientation	16. I am generally satisfied with my performance at work	A3
**Physical Health**	Biological	17. I typically feel physically healthy	H1
		18. I am rarely sick	H2
	Functional	19. I can typically overcome sources of physical distress (e.g., insomnia, injuries, and vision issues)	H3
	Psychological	20. I feel in control of my physical health	H4
**Mindset**	Growth Mindset	21. I believe I can improve my job skills through hard work	MI1
	Prospection	22. I believe my job will allow me to develop in the future	MI2
		23. I have a bright future at my current work organization	MI3
**Environment**	Physical	24. My physical work environment (e.g., office space) allows me to focus on my work	EN1
		25. There is plenty of natural light in my workplace	EN2
		26. I can conveniently access nature in my work environment (e.g., parks, oceans, and mountains)	EN3
**Economic Security**	Income	27. I am comfortable with my current income	ES1
	Medical Spending	28. I could lose several months of pay due to serious illness, and still have my economic security	ES2
	Financial Savings	29. In the event of a financial emergency, I have adequate savings	ES3

Response set ranged from 1 (Strongly Disagree) to 7 (Strongly Agree).

Second, the PFW Scale has been found to predict essential work outcomes, such as turnover intentions, job-related affective wellbeing, plus individual, team, and organizational adaptivity, proactivity, and organizational proficiency (Donaldson and Donaldson, 2021a), as well as academic success (Weiss et al., 2021). Therefore, it is a comprehensive measurement tool that can help determine the needs of students, workers, leaders, and organizations and can be used to guide the design and evaluate POP interventions (Donaldson and Chen, 2021).

Third, to examine if common research biases might have inflated estimates of the PERMA and PERMA+4 in their relationship to wellbeing, three rigorous multi-trait multi-method (MTMM) analyses with 220 knowledgeable co-worker pairs (*N* = 440) were recently carried out. Initially, Donaldson et al. (2020) found that the original 5 PERMA building blocks (positive emotions, engagement, relationships, meaning, and accomplishment) and the four additional potential building blocks of PERMA+4 (physical health, mindset, environment, and economic security) significantly predicted life satisfaction above and beyond self-report and mono-method bias. Next, Donaldson et al. (2021) extended this line of MTMM research and found strong support for the validity of the relationship between overall PERMA+4 and work role performance, including adaptivity, proactivity, and proficiency after correcting for self-report and mono-methods bias. A third analysis was conducted to understand one of the nine PERMA+4 building blocks in depth, namely positive mindset as measured by psychological capital – Hope, Efficacy, Resilience, and Optimism (HERO; Donaldson et al., 2021). Positive Mindset (PsyCap) was also found to be a strong predictor of work role performance above and beyond self-report and mono-method bias (Donaldson et al., 2021). Donaldson et al., 2020 also found that this building block of positive mindset (HERO) predicted work role performance for 3,860 employees across 15 nations. These rigorous MTMM analyses combined with the other primary and large meta-analytic studies presented in this paper strongly suggest the PERMA+4 framework could be a promising way to organize future research and guide the design and evaluation of future interventions in POP 2.0.

## Future Perspectives: PERMA+4 and POP 2.0

Research in POP has shown exponential growth over the past 5 years (Martín-del-Río et al., 2021). This exponential growth may indicate that the discipline is on the horizon of a new wave of research, innovation and ideas, which may fundamentally alter its discourse. Two recent studies have further solidified the evidence showing the strong association between wellbeing and performance at work, the targets of the PERMA+4 building blocks. First, Moscoso and Salgado (2021) meta-analyzed the relationship between well-being and work performance with a database of 34 independent samples (*n* = 5352) using supervisory performance ratings and 38 independent samples (*n* = 12086) using self-reported of job performance. The findings revealed a substantial correlation across all the wellbeing measures used (overall subjective, affective, and cognitive wellbeing) with supervisory performance ratings and self-reported performance. Next, Lester et al. (2021) examined the prediction of affective wellbeing to work performance in a sample of 908,096 US Army soldiers (with over ¼ of a million ethnic minorities and over 150,000 women). It was found that wellbeing measures predicted awards for outstanding performance over a four-year follow-up period, in which 114,443 soldiers (12.60%) received an award. Furthermore, each wellbeing variable predicted future awards for both women and men, for enlisted soldiers as well as officers, for several ethnicities, for varying levels of education, and controlling for several other potential explanatory variables. These new studies provide additional compelling evidence supporting the link between work-related wellbeing and work performance.

Another important line of work likely to improve and expand during POP 2.0 is generally known as positive approaches to diversity, equity, and inclusion (DEI; see Rao and Donaldson, 2015; Warren et al., 2019). Donaldson et al. (2021) recently systematically reviewed and analyzed the findings from 25 meta-analyses, 42 review papers, and hundreds of high-quality randomized controlled trials of Positive Psychology Interventions (PPIs) designed to generate wellbeing. In addition, to identifying and analyzing the most exemplary PPIs with an eye toward improving the design of the next-generation of PPIs (Donaldson and Chen, 2021), they found most PPIs have been primarily studied in western, educated, industrial, rich, democratic (WEIRD) countries. One conclusion they reached is more rigorous research on PPIs serving diverse populations and in non-WEIRD contexts is needed to ensure equitable access to effective interventions that generate wellbeing for all. Warren et al. (2019) have suggested a framework to guide these future DEI efforts, and Donaldson and Chen (2021) have provided examples of what new PPIs focused on DEI topics, such as cultural humility at work and a positive approach to preventing sexual harassment in the workplace, could look like in POP 2.0.

We expect to see a new wave of research in the coming years that will include topics like social and organizational network analysis of positive leadership and relational energy in the workplace and more advances in artificial intelligence-driven positive organizational interventions, human-robot collaboration, passive neurological assessments of positive states/traits and behaviors and the like (e.g., see Margolis et al., 2021). This new wave of research will be categorized by rapid innovation, mass adoption of artificial intelligence systems, machine learning, social media analytics, big data analyses, and the like that we will learn from immensely during POP 2.0. These rapid changes will also require more sophisticated models, approaches, and measures which could stand the test of time; yet are flexible to adapt to new innovations and discourses in technology and the discipline. We therefore propose that the PERMA+4 could be used as one of the first models to drive innovation in the wellbeing and sustainable work performance space for POP 2.0.

While evidence into the effectiveness of the PERMA+4 approach has shown promise as a means to predict wellbeing and work performance, research is still in its infancy. To further introduce such into the nomological network of POP 2.0, more research is required into its antecedents/outcomes, how it is measured/approached, and how PERMA+4 can be developed.

### PERMA+4: Outcomes and Antecedents

PERMA+4 is positioned as a framework describing the routes towards work-related wellbeing and performance at work. In essence, it implies that PERMA+4 could be used as a process model or framework that could translate important antecedents into wellbeing and performance. Therefore, it is imperative for future research to systematically contrast and compare different input factors (such as work role fit, psychological safety/availability, and job crafting) to determine the most important antecedents for the PERMA+4 building blocks (Donaldson and Chen, 2021). Through identifying the most important antecedents, researchers and practitioners could build more robust and concrete interventions. Further, a major point of contention within the wellbeing literature is the role of signature or “psychological strengths” in the development of wellbeing (van Zyl et al., 2021). Theory argues that strengths-presence and strengths-knowledge are integral for wellbeing; however, only active strength use has shown to be an essential wellbeing and performance metric. Given that strengths are central to the developing metatheory of positive psychology, it is essential to understand and investigate its role in enhancing work-related wellbeing and performance, and what the role of PERMA+4 is to translate strengths-presence, − knowledge and use into sustainable mental health. Future research should position PERMA+4 as a process factor, and not an active or targeted antecedent of wellbeing. Therefore, focus should be on “what factors are needed to activate PERMA+4 as a means to enhance work-related wellbeing and work performance.” Further, the specific individual, group or team, and organizational related outcomes of PERMA+4 (above and beyond wellbeing or mental health) should be investigated. This would not only provide the literature with more support for its effectiveness but provide a solid business case for its active incorporation into HRD practices in industry. Here, focus should be on linking the PERMA+4 to objective strategic growth indicators or to the financial performance of organizations.

### The Measurement of PERMA+4

Effective measurement is a central component to the advancement of a discipline and the development of theory. The PFW Scale is a relatively newly developed psychometric instrument aimed at measuring the building blocks of wellbeing. However, despite the robust approach employed in its development, there are still many questions and concerns that need further exploration. First, the instrument was developed within a strictly western context and its cross-cultural equivalence is therefore required. Therefore, the PFW Scale should be subjected to more robust validation processes, with more diverse samples, from different cultural groups and nationalities to determine its viability as a measure. Second, the length of the current instrument increases the possibility for common method bias, acquaintance bias, and measurement error (Peytchev and Peytcheva, 2017). Lengthy self-report questionnaires are known to produce to cause response fatigue, which negatively impacts on the quality of the data (Peytchev and Peytcheva, 2017; Andreadis and Kartsounidou, 2020). Further, the length of a questionnaire also impacts the response rate, dropouts and overall response quality (Andreadis and Kartsounidou, 2020). Therefore, future psychometric evaluations of the P-F Work Scale should be directed toward significantly shortening the scale.

Third, another area to consider in the measurement of PERMA+4, is to assess work-related wellbeing and performance from a physiological and behavioral perspective. In their position paper, Cipresso and Immekus (2017) argued that psychological researchers should move away from self-report measures and include more objective indicators for their assessments of (positive) psychological states, traits and behaviors. Drawing from advancements in measurement methodology, we believe future developments in the assessment of PERMA+4 could complement self-report measures with biosensors. This will allow, for example, for the uninterrupted measurement of the PERMA+4 components during an intervention without interruption. By incorporating superficial electromyography assessments into the measurement, approaches would allow researchers to passively assess wellbeing indicators such as positive emotions and engagement through facial muscle activation. Other psychophysiological responses associated with wellbeing could also be assessed through wearable technologies. Here, smart watches, for example, could be used to measure cardiovascular activity, respiration, respiratory inductance plethysmography (through thoracic strips), blood oxygen saturation, and the like could be used as indicators for positive emotions, engagement, and physical health. Neuro imaging could also be used to assesses experiences of accomplishments and the neurophysiological responses associated with building positive relationships (Cipresso and Immekus, 2017). Psychophysiological responses associated with experiences of PERMA+4 could also be captured through measuring hormones (such as cortisol levels; Vázquez et al., 2009; Lazzarino et al., 2013).

From an (objective) behavioral assessment perspective, it is important to investigate if what people self-report on PERMA+4 and how they behave are aligned. Technology could close the gap between what people think they feel or perceive and what they actually perceive (Cipresso and Immekus, 2017). We suggest that future researchers invest in developing activity-related behavioral assessment measures whereby wellbeing could objectively be assessed through the language people use, the physical expression, voice tones, postures, gestures, body movement, and the like. These aspects are already used as indicators for mental illness assessments and could easily be adapted to measure mental health. Sport psychology and health psychology interventions already employ motion sensors, accelerometers, and gyroscopes in modern cellphones as indicators of physical and mental health (Cipresso and Immekus, 2017). We see scope for expanding their use into organizational contexts through assessing PERMA+4.

Fourth, we suggest that latent profile analysis be used in conjunction with computer-adaptive assessments, in order to determine and diagnose the “type” of profiles people exhibit in their pursuits to enhance their wellbeing. This would aid in creating more tailored intervention strategies which are more aligned to the needs, wants and strengths of participants. Further, by using computer-adaptive assessments, more accurate profiling can be done with a lot fewer items. Finally, future research should further investigate the construct validity of the PERMA+4 model and the associated PFW Scale. Donaldson (2019) and Donaldson et al. (2020) have already demonstrated that the PFW Scale is related to other scales such as psychological capital (Luthans et al., 2007) and life satisfaction (Diener et al., 1985). Future investigations should aim to relate the scale to other work-related wellbeing measures (e.g., Flourishing at work Scale; Rothmann et al., 2019) and work performance (e.g., Individual Work Performance Scale; Koopmans et al., 2013) to ensure that it does, indeed, behave how the theory states it should. In summation, the measurement of PERMA+4 should take central stage in future research.

### Developing PERMA+4

The PERMA+4 model is positioned as a roadmap for factors leading to work-related wellbeing and sustainable work performance. Although research has shown that the individual factors of the approach are strongly related to wellbeing and work performance, evidence as to the practical usefulness thereof is still lacking. Multi-component positive psychological interventions are therefore needed (built around each component of the PERMA+4 model) in order to determine if these routes toward wellbeing and work performance are, indeed, relevant in practice. It is therefore important to investigate how interventions could improve each of the building blocks in PERMA+4 and which are more efficient in enhancing wellbeing and work performance at the employee, leadership, group or teams, and organizational levels (see Donaldson and Chen, 2021). Further, technologically driven intervention strategies should also take center stage in future research.

Given the rapid rise and adoption of artificial intelligence (AI) in psychology, we expect to see a rise in AI-driven positive psychological interventions within organizations ranging from AI-Coaching to AI-driven chat bots aimed at enhancing wellbeing (Greer et al., 2019; Worthington and van Zyl, 2021). Fully automated conversation agents (or “chat bots”) could automate the diagnosis of current challenges and generate appropriate self-help interventions tailored to the needs of the individuals (Greer et al., 2019). These chat bots do not require active input from a therapist, coach, or practitioner, enhancing its perceived accessibility and usefulness. Therefore, allowing for intervention content to be generated and used when it is needed and eliminates the delay between the experience of a problem and a potential solution (Greer et al., 2019). The use of chat bots is still rare within organizational contexts but will become increasingly important over the next two decades (Laranjo et al., 2018). Further, virtual reality or augmented-related interventions could be used to facilitate the development of positive states, traits, and behaviors through an immersive environment which is tailored to the needs/circumstances/context of the client (Baños et al., 2014, 2021). Video games could also be used as a safe and cost-effective means to develop wellbeing and enhance performance (Kelly, 2020; Baños et al., 2021). Kelly (2020) argued that video games are naturally designed to enhance the core capabilities known to enhance wellbeing, such as creativity, pleasure, engagement, meaning, social skills, emotional regulation, attention, environmental mastery, accomplishments (through skill progression) and also affords individuals the opportunities to live out their strengths in a safe environment.

## Conclusion

The evidence accumulated by POP over the past two decades strongly supports the link between wellbeing and performance at work and that such could effectively be developed through POP interventions. PERMA+4 might be used as one framework to guide future efforts to build the evidence-base for the science of POP. It could also be used as a framework to guide educational efforts, consulting and coaching protocols, and next-generation POPIs, in what we might imagine could go down in history as the second phase of research and practice known as POP 2.0.

## Data Availability Statement

The original contributions presented in the study are included in the article/supplementary material, and further inquiries can be directed to the corresponding author.

## Author Contributions

All authors listed have made a substantial, direct and intellectual contribution to the work, and approved it for publication.

## Conflict of Interest

The authors declare that the research was conducted in the absence of any commercial or financial relationships that could be construed as a potential conflict of interest.

## Publisher’s Note

All claims expressed in this article are solely those of the authors and do not necessarily represent those of their affiliated organizations, or those of the publisher, the editors and the reviewers. Any product that may be evaluated in this article, or claim that may be made by its manufacturer, is not guaranteed or endorsed by the publisher.
